# Epidemiological trend in scarlet fever incidence in China during the COVID-19 pandemic: A time series analysis

**DOI:** 10.3389/fpubh.2022.923318

**Published:** 2022-12-15

**Authors:** Yunxia Ma, Shanshan Gao, Zheng Kang, Linghan Shan, Mingli Jiao, Ye Li, Libo Liang, Yanhua Hao, Binyu Zhao, Ning Ning, Lijun Gao, Yu Cui, Hong Sun, Qunhong Wu, Huan Liu

**Affiliations:** ^1^Department of Social Medicine, Health Management College, Harbin Medical University, Harbin, China; ^2^Department of Biostatistics, School of Public Health, Harbin Medical University, Harbin, China

**Keywords:** COVID-19, scarlet fever, SARIMA, TBATS, non-pharmaceutical interventions, ITSA

## Abstract

**Objective:**

Over the past decade, scarlet fever has caused a relatively high economic burden in various regions of China. Non-pharmaceutical interventions (NPIs) are necessary because of the absence of vaccines and specific drugs. This study aimed to characterize the demographics of patients with scarlet fever, describe its spatiotemporal distribution, and explore the impact of NPIs on the disease in the era of coronavirus disease 2019 (COVID-19) in China.

**Methods:**

Using monthly scarlet fever data from January 2011 to December 2019, seasonal autoregressive integrated moving average (SARIMA), advanced innovation state-space modeling framework that combines Box-Cox transformations, Fourier series with time-varying coefficients, and autoregressive moving average error correction method (TBATS) models were developed to select the best model for comparing between the expected and actual incidence of scarlet fever in 2020. Interrupted time series analysis (ITSA) was used to explore whether NPIs have an effect on scarlet fever incidence, while the intervention effects of specific NPIs were explored using correlation analysis and ridge regression methods.

**Results:**

From 2011 to 2017, the total number of scarlet fever cases was 400,691, with children aged 0–9 years being the main group affected. There were two annual incidence peaks (May to June and November to December). According to the best prediction model TBATS (0.002, {0, 0}, 0.801, {<12, 5>}), the number of scarlet fever cases was 72,148 and dual seasonality was no longer prominent. ITSA showed a significant effect of NPIs of a reduction in the number of scarlet fever episodes (β2 = −61526, *P* < 0.005), and the effect of canceling public events (c3) was the most significant (*P* = 0.0447).

**Conclusions:**

The incidence of scarlet fever during COVID-19 was lower than expected, and the total incidence decreased by 80.74% in 2020. The results of this study indicate that strict NPIs may be of potential benefit in preventing scarlet fever occurrence, especially that related to public event cancellation. However, it is still important that vaccines and drugs are available in the future.

## 1. Introduction

*Streptococcus pyogenes* (group A *Streptococcus*, GAS) is the most virulent of all clinically important streptococci and is considered the fifth most deadly pathogen globally, posing not only a major threat to humans but also a heavy global disease burden (1–3). One study estimated that at least half a million people worldwide die each year from severe GAS infection (4). Scarlet fever is an acute respiratory infection caused by GAS and is usually spread by respiratory droplets or direct contact with mucus, saliva, or the skin of an infected person (5, 6). Although scarlet fever is a benign infectious disease, it is prone to clusters of outbreaks accompanied by complications including otitis media, pneumonia, and sepsis, which causes a constant drain on healthcare resources (7, 8). In the mid-19th century, scarlet fever was a major cause of death among children worldwide (9, 10). By the 20th century, the mortality rate had decreased considerably due to improved sanitation and widespread use of effective antibiotics (9, 11). Since the 21st century, the re-emergence of scarlet fever has become a major public health concern in several countries and regions. In 2011, the incidence of scarlet fever increased rapidly in South Korea (12). In addition, in 2014, the incidence of scarlet fever in the United Kingdom reached a new highest level in 50 years (13).

Scarlet fever was classified as a category B notifiable disease in China in 1950 and caused a significant economic burden in the early 1980s, after which the incidence gradually declined (14). The disease returned in 2011, associated with the rapid economic development, living standards, population mobility, and host population genetics in China (5). One study confirmed that the average annual incidence of scarlet fever in China was twice as high between 2011 and 2016 as that before between 2004 and 2011 (15). This may be closely related to the national policy of liberalizing the second child, which puts the population of children susceptible to scarlet fever at great risk (16). The preventive management of scarlet fever in China should increase to a new level. However, there are relatively few studies on scarlet fever in China, which mainly focus on specific regions or cities, and the results of these studies may be diverse, fragmented, and inconclusive. Therefore, a comprehensive and systematic study of scarlet fever in mainland China is needed (6, 17). In addition, there is still no effective vaccine available for preventing scarlet fever; therefore, the importance of effective non-pharmaceutical interventions (NPIs) should be emphasized.

The coronavirus disease 2019 (COVID-19) pandemic has been spreading since the end of 2019. To contain the spread of the epidemic in a timely and effective manner, governments have actively adopted NPIs such as masking, lockdown policies, and distancing (18–21). Results from a global study noted that a number of NPIs reduced the time-varying reproduction number of COVID-19 by 3–24% by day 28 after introduction (22). As the first epicenter of COVID-19, the epidemic was effectively controlled in China after the adoption of strict NPIs. A study found that the proportion of serious and critical COVID-19 cases fell from 53.1 to 10.3% in the 3 months following the implementation of NPIs (23). It has also been found that NPIs have a positive effect on the prevention and control of respiratory infections (24–26). To our knowledge, few studies have been conducted, using quantitative analysis, on the effect of NPIs for a specific disease, such as scarlet fever, during the COVID-19 era. In addition, the rigorous NPIs adopted in China may help in studying changes in the incidence of scarlet fever during COVID-19. Thus, a robust and accurate predictive model, which is important for predicting the incidence of scarlet fever in the COVID-19 era, is needed to detect and analyze trends during this period.

Many forecasting methods have been widely adopted as effective policy support tools to assess and analyze the temporal patterns of infectious disease incidence, among which the autoregressive integrated moving average (ARIMA) model has proven to be more effective (27–29). Research has shown that scarlet fever has multiple seasonal patterns (15), and the advanced innovation state-space modeling framework that combines Box-Cox transformations, Fourier series with time-varying coefficients, and autoregressive moving average (ARMA) error correction method (TBATS) model works better in dealing with complex time series analyses with seasonal cycles, non-integer seasonality, and dual calendar effects (30). To the best of our knowledge, few studies have used these advanced methods to analyze and assess the long-term epidemiological trends and seasonality of scarlet fever. To demonstrate their applicability, the level of precision was compared using an ARIMA model with seasonality (SARIMA).

In summary, this study aimed to examine the demographic and spatiotemporal distribution and characteristics of scarlet fever re-emergence in mainland China from 2011 to 2017. We also verified whether the adoption of strict NPIs in China, had an impact on the incidence of scarlet fever in the COVID-19 era, and which specific measures had the greatest impact. The findings might provide evidence and support for future scarlet fever prevention and control.

## 2. Materials and methods

### 2.1. Ethical statement

Pooled data were obtained from publicly available monitoring platforms and ethical approval or informed consent was considered unnecessary.

### 2.2. Data collection

Scarlet fever is a nationally notifiable infectious disease in China. The scarlet fever data used in this study were obtained from two main sources. (1) Data on the regional distribution and demographic characteristics of scarlet fever in mainland China were extracted from the China Public Health Science Data Center (https://www.phsciencedata.cn/Share/index.jsp)^1^. As the latest demographic data were only updated until 2017, only the data in the years 2011–2017 were included in the preliminary descriptive analysis of the demographics. (2) Monthly data of new cases of scarlet fever in China from January 1, 2011 to December 31, 2020, were collected from the National Heath Commission of the People's Republic of China (http://www.nhc.gov.cn/wjw/index.shtml)^2^. This study constructed SARIMA and TBATS models using the 2011–2018 data, and evaluated the predictive effect of the models using the 2019 data.

The source of data for comprehensive NPIs in the interrupted time series analysis (ITSA) is the Oxford COVID-19 Government Response Tracker (OxCGRT), which was developed by Oxford scholars in 2020 to track the government's response to the coronavirus pandemic. In addition, this study conducted analysis on the following eight specific NPIs included in containment and closure policies in the OxCGRT: school closures (c1), workplace closures (c2), cancellation of public events (c3), restrictions on public gatherings (c4), closures of public transport (c5), stay-at-home requirements (c6), restrictions on internal movements (c7), and international travel controls (c8). The data for each NPIs are from Our World in Data (https://ourworldindata.org/covid-stringency-index)^3^.

### 2.3. TBATS model

Traditional seasonal exponential smoothing methods cannot be used to describe complex seasonal time series including multiple and non-integer seasonal patterns. The BATS (*p, q*, *m*_1_, *m*_2_, ..., *m*_T_) method is thus proposed, where B represents the Box-Cox conversion, A represents the ARMA model, and *T* and *S* represent the trend and seasonal patterns in the target series, respectively (31, 32). The key parameters of the BATS model are the ARMA method (*p* and *q*) and the seasonal period (*m*_1_, ..., *m*_T_). The advanced TBATS (ω, *p, q*, ϕ, {*m*_1_, *k*_1_}, {*m*_2_, *k*_2_} ..., {*m*_T_, *k*_T_}) model was developed by adding a Fourier series-based trigonometric representation of the seasonal components to the traditional BATS method, which can handle complex time series as well as linear and non-linear time series (33) while adapting to dynamic seasonal patterns over time (30). The parameters (*p, q*, and *m*) of the TBATS model are consistent with those of the BATS model, where *k* is the number of corresponding Fourier terms used for each seasonality, ω is the Box-Cox transformation, and ϕ is the damping parameter that facilitates trend extrapolation to the model when the trend pattern is weakened (31, 34). The TBATS model has many parameters, and this study automated the determination of the values of each parameter in R software using the principle of Akaike information criterion (AIC) minimization to fit the model. It is worth mentioning that the TBATS model has the potential to decompose the time series into different components, enabling the identification and extraction of one or more seasonal features that may not be present in the object series graphs.

### 2.4. SARIMA model

The ARIMA model is a classical time-series predictive analysis method proposed by Box and Jenkins, which is mainly used to fit time series that are stationary (or can be converted to stationary). Scarlet fever frequently has notable seasonal effects (29), hence the use of the SARIMA method. The SARIMA (*p, d, q*) (*P, D, Q*) model is based on the ARIMA model (27). In this method, the seasonality of scarlet fever was considered as the explanatory variable while the monthly scarlet fever incidence was the response variable, and the model's equation is


(1) $$\left\{ \begin{array}{l} \varphi \left(B\right)\Phi \left(B^{s}\right)\Delta^{d}\Delta_{s}^{D}X_{t}=\theta \left(B\right)\Theta \left(B^{s}\right)\varepsilon_{t}\\ E\left(\varepsilon_{t}\right)=0,Var\left(\varepsilon_{t}\right)=\sigma_{\varepsilon }^{2},E\left(\varepsilon_{t}\varepsilon_{s}\right)=0,s\neq t\\ \;E\left(X_{s}\varepsilon_{t}\right)=0,\forall_{s}<t \end{array}\right.$$

where *B* indicates the backward shift operator, ε_*t*_signifies the errors of prediction, *S* denotes the periodicity of the scarlet fever incidence series (*S* = 12 in this study), while *d* and *D*, are the non-seasonal and seasonal differences in times, respectively. *p* and *q* are the orders of the autoregressive and moving average models, respectively. *P* and *Q* are the orders of the seasonal autoregressive and moving average models, respectively. This study used an automated time-series modeling for the specified sample data.

Building the SARIMA model followed these key steps: First, the stationarity of the scarlet fever incidence series was examined using the Augmented Dickey-Fuller (ADF) method (35). When the result of the ADF test was significant, the sequence was proved to be stationary. For the non-stationary scarlet fever series, log transformation or differencing was adopted to fulfill the stationarity assumption. Second, an autocorrelation function (ACF) graph and partial autocorrelation (PACF) plots were used to select reasonable parameters for the SARIMA model (36). Meanwhile, the auto.arima function of R 4.1.1 software had been used to select a best SARIMA model according to either the minimum of the AIC, AICc, or BIC. Third, we evaluated the fit of the model to make predictions. Generally, if a model was appropriate, the residuals of the model should meet the independent distribution assumption; that is, there was no correlation between the residuals. Finally, the residual was determined as a white noise series using the Ljung-Box *Q* test (37).

### 2.5. Performance statistics index

To assess the accuracy of the model predictions, two metrics, the root mean square error (RMSE) and mean absolute percentage error (MAPE), were used to compare the forecasting capabilities of the TBATS and SARIMA models. The smaller the measure, the better the corresponding model. The calculation formula is as follows:


(2) $$\begin{array}{l} RMSE=\sqrt{\frac{1}{n}{\sum_{t=1}^{n}\left(y_{t}-{ŷ}_{t}\right)}^{2}} \end{array}$$

(3) $$\begin{array}{l} MAPE=\frac{1}{n}\sum_{t=1}^{n}\frac{\left|y_{t}-{ŷ}_{t}\right|}{y_{t}} \end{array}$$

### 2.6. Statistical analysis

First, we conducted a descriptive analysis of the demographic and spatiotemporal distribution of scarlet fever incidence in mainland China from 2011 to 2017. Second, the TBATS and SARIMA models were evaluated using two indicators, RMSE and MAPE, to select the best model to predict scarlet fever incidences in 2020 and to observe the changes in the actual and expected number of cases. Finally, this study used ITSA to explore whether comprehensive NPIs had an effect on the number of cases and further analyzed which specific NPIs had a significant effect on scarlet, using correlation analysis and ridge regression. It is worth noting that each NPI must be standardized prior to the statistical analysis.

Multiple statistical packages including “forecast,” “tseries,” and “tbats” in R (version 4.1.1, R Development Core Team, Vienna, Austria) were employed to establish the SARIMA and TBATS models. All the estimated parameter values were statistically significant (*P* < 0.05). In addition, statistical R packages such as “prais” and “sandwich” were used for the ITSA. Correlation and ridge regression analyses were performed using IBM SPSS Statistics for Windows version 24.0 (IBM Corp., Armonk, NY, USA).

## 3. Results

### 3.1. Demographic and distributive features of scarlet fever from 2011 to 2017

The characteristics of the patients with scarlet fever in mainland China are shown in Table 1. From 2011 to 2017, 400,691 cases of scarlet fever were reported in mainland China, with an average of 57,000 cases per year and the highest incidence occurred in 2017. More than half of all patients were males, with a male-to-female ratio of 1.59:1. Second, the majority of the patients were children aged 0–9 years (92.83%). In addition, kindergarten children, students, and scattered children were the main groups diagnosed with scarlet fever between 2011 and 2017.

**Table 1:** Demographic and distributive features of scarlet fever in mainland China from 2011 to 2017.

**Variable**	**Total**	**2011**	**2012**	**2013**	**2014**	**2015**	**2016**	**2017**
	***N* = 400,691 (%)**	***n* = 63,878 (%)**	***n* = 46,459 (%)**	***n* = 34,207 (%)**	***n* = 54,247 (%)**	***n* = 68,249 (%)**	***n* = 59,282 (%)**	***n* = 74,369 (%)**
**Sex**								
Male	245,777 (61.34)	39,826 (62.35)	29,039 (62.50)	20,736 (60.62)	32,932 (60.71)	41,502 (60.81)	36,390 (61.38)	45,352 (60.98)
Female	154,914 (38.66)	24,052 (37.65)	17,420 (37.50)	13,471 (39.38)	21,315 (39.29)	26,747 (39.19)	22,892 (38.62)	29,017 (39.02)
**Age, years**								
0–9	371,968 (92.83)	58,825 (92.09)	41,723 (89.80)	31,268 (91.41)	50,348 (92.81)	63,963 (93.72)	55,589 (93.77)	70,252 (94.46)
10–19	24,727 (6.17)	4,401 (6.89)	4,082 (8.79)	2,468 (7.21)	3,321 (6.12)	3,710 (5.44)	3,147 (5.31)	3,598 (4.84)
20–29	2,440 (0.61)	437 (0.68)	436 (0.94)	287 (0.84)	357 (0.66)	335 (0.49)	319 (0.54)	269 (0.36)
30–39	914 (0.23)	112 (0.18)	125 (0.27)	114 (0.33)	135 (0.25)	137 (0.20)	139 (0.23)	152 (0.20)
40–49	346 (0.09)	57 (0.09)	52 (0.11)	34 (0.10)	51 (0.09)	55 (0.08)	43 (0.07)	54 (0.07)
50–59	170 (0.04)	27 (0.04)	22 (0.05)	25 (0.07)	20 (0.04)	31 (0.05)	28 (0.05)	17 (0.02)
≥60	126 (0.03)	19 (0.03)	19 (0.04)	11 (0.03)	15 (0.03)	18 (0.03)	17 (0.03)	27 (0.04)
**Occupation**								
Children in kindergarten	168,601 (42.08)	25,357 (39.70)	18,956 (40.80)	13,303 (38.89)	21,476 (39.59)	29,014 (42.51)	26,283 (44.34)	34,212 (46.00)
Students	151,778 (37.88)	25,809 (40.40)	17,958 (38.65)	13,352 (39.03)	21,912 (40.39)	25,299 (37.07)	21,195 (35.75)	26,253 (35.30)
Scattered children	75,810 (18.92)	11,822 (18.51)	8,698 (18.72)	7,040 (20.58)	10,252 (18.90)	13,337 (19.54)	11,251 (18.98)	13,410 (18.03)
Farmers	1,503 (0.38)	246 (0.39)	257 (0.55)	196 (0.57)	214 (0.39)	234 (0.34)	188 (0.32)	168 (0.23)
Housework and unemployment	903 (0.23)	142 (0.22)	150 (0.32)	101 (0.30)	143 (0.26)	127 (0.19)	99 (0.17)	141 (0.19)
Workers	455 (0.11)	80 (0.13)	78 (0.17)	60 (0.18)	71 (0.13)	60 (0.09)	65 (0.11)	41 (0.06)
Others	1,641 (0.41)	422 (0.66)	362 (0.78)	155 (0.45)	179 (0.33)	178 (0.26)	201 (0.34)	144 (0.19)

### 3.2. Spatiotemporal analyses

**Figure 1:**
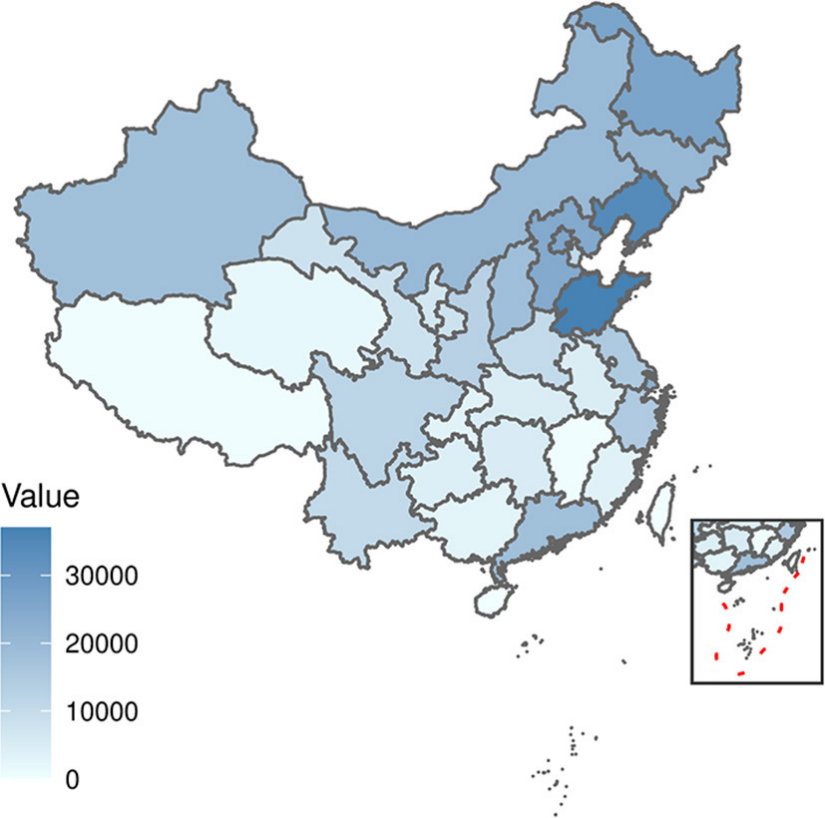
Distribution of the total number of scarlet fever cases in each province in China from 2011 to 2017.

**Figure 2:**
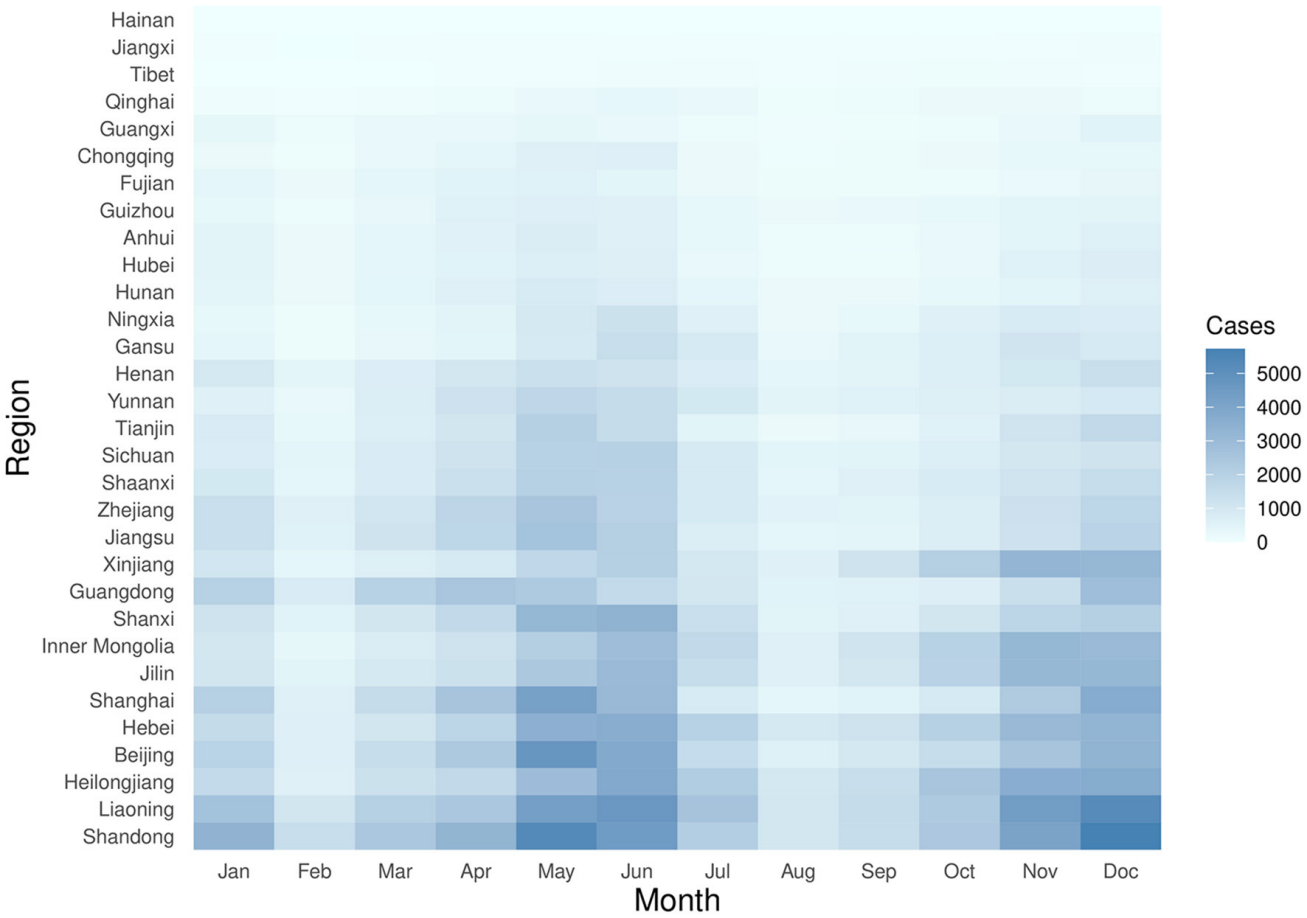
The time series of monthly number of cases from 2011 to 2017, standardized by the monthly number of cases reported by each province according to the total number of scarlet fever cases recorded in the 7 years.

The distribution by the total number of scarlet fever and by province in China from 2011 to 2017 revealed a high degree of dispersion (Figure 1). Northern regions such as Shandong, Liaoning, and Heilongjiang had a high incidence of scarlet fever. In contrast, the incidence was very low in areas such as Hainan and Tibet. Based on the monthly number of reported cases by province in China from 2011 to 2017 and ranked by the total number of scarlet fever cases over the seven years, Shandong and Liaoning showed the most prominent incidence rates. Among the months observed, the incidence was highest in May, June, November, and December, and the highest incidence in December was observed in Shandong Province (Figure 2).

**Figure 3:**
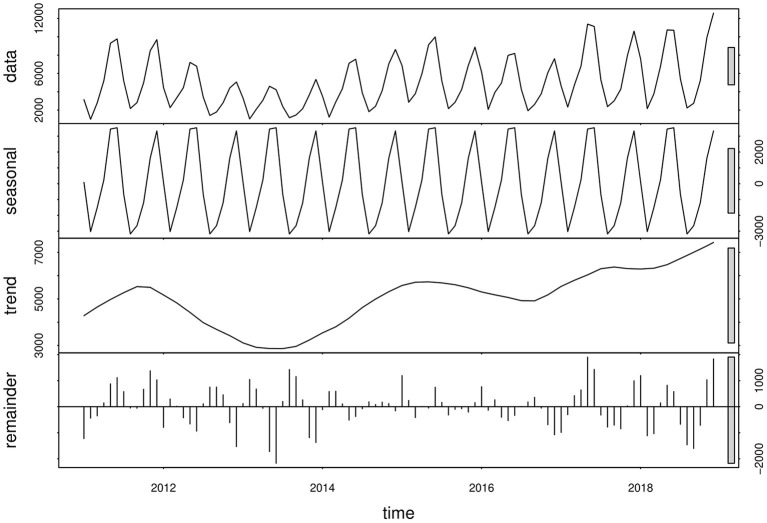
Monthly scarlet fever incidence and variations, from January 2011 to December 2018.

A clear cyclical and seasonal pattern of monthly scarlet fever incidence and trend was observed from January 2011 to December 2018 (Figure 3). Since 2013, the incidence of scarlet fever in mainland China has been fluctuating and increasing, and has continued to show an increasing trend in recent years.

### 3.3. Sample simulation and prediction

**Figure 4:**
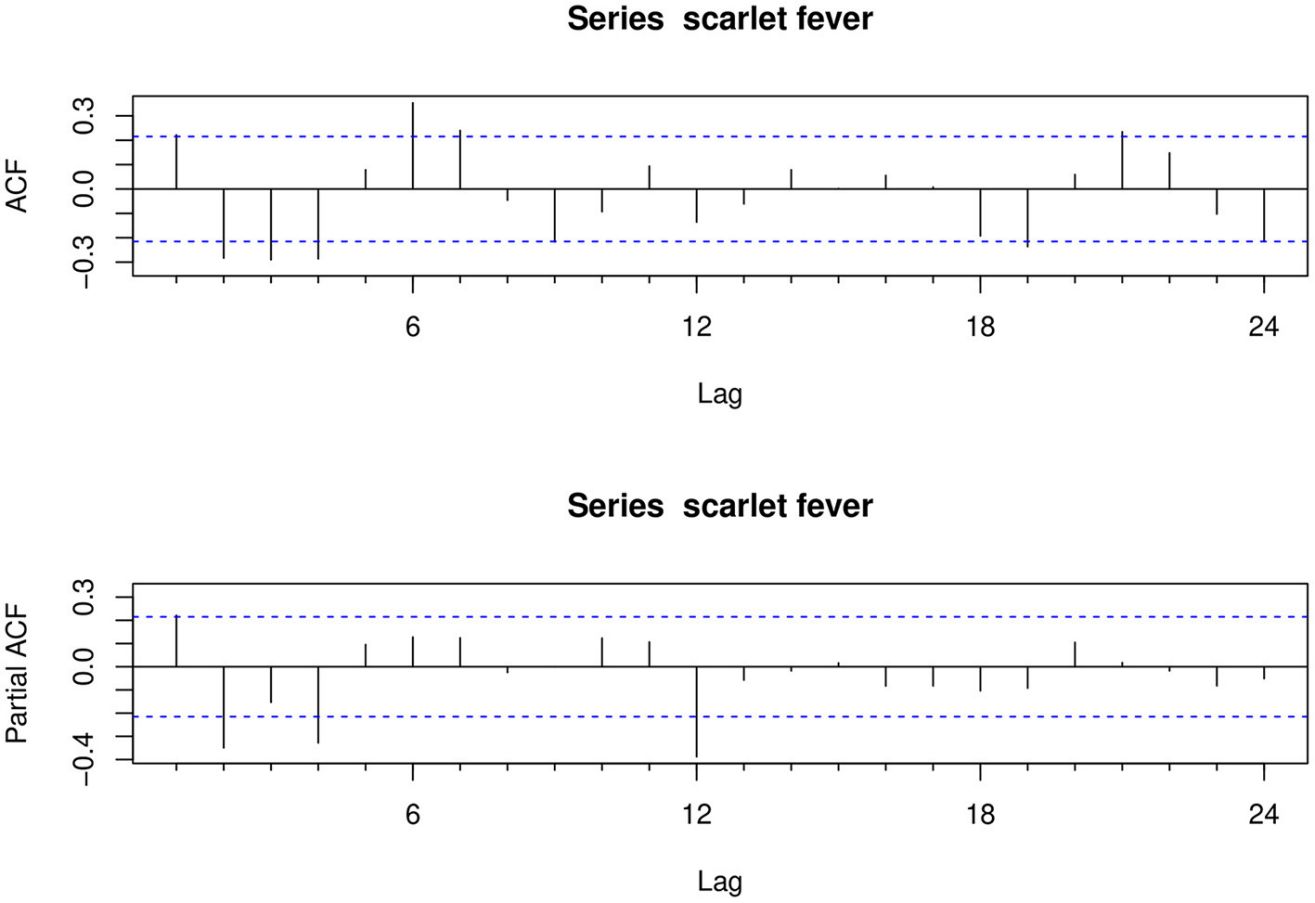
Autocorrelation and partial autocorrelation plots for the differenced scarlet fever time series.

The ndiffs show that the scarlet fever time series was not smooth; first-order difference (*d* = 1) and seasonal difference (*D* = 1) were determined, and the final ADF test was statistically significant (*P* < 0.01), making the series smooth. The ACF and PACF graphs (Figure 4) were generated to help estimate the other parameters. The model automatically selected SARIMA (2, 1, 2) (0, 1, 1)_[12]_ as the best fit [AIC = 1,356.48, Bayesian information criterion (BIC) = 1370.99] using time-series modeling software on the specified sample data. Therefore, in this study *p, d, q* = 2, 1, 2 and *P, D, Q* = 0, 1, 1, respectively. The Ljung-Box *Q* test further indicated that the model residuals were consistent with the white noise series (χ^2^ = 0.0018521, *P* > 0.05), indicating that the residual series was purely random and that the SARIMA model extracted sufficient information. In addition, sensitivity analyses were constructed by varying *p, q* in SARIMA. Based on similar studies where the range of *p, q* should not be too large, the range of values for *p, q* in this study was 0–3. The results of the sensitivity analysis (Supplementary Table 1) imply that the SARIMA (2, 1, 2) (0, 1, 1)_[12]_ model identified can effectively and adequately track the epidemiological trends of scarlet fever in mainland China. The TBATS model included numerous parameters and was automatically modeled in *R* using the “tbats” function to obtain the model parameters ω =0.003, ϕ = 0.822, *p* = 0, *q* = 0, seasonal cycle length *m*_T_ = 12, *k*_T_ = 5, and model AIC = 1716.454. A final TBATS model (0.003, {0, 0}, 0.822, { <12, 5>}) was obtained.

### 3.4. Model performance evaluation

**Figure 5:**
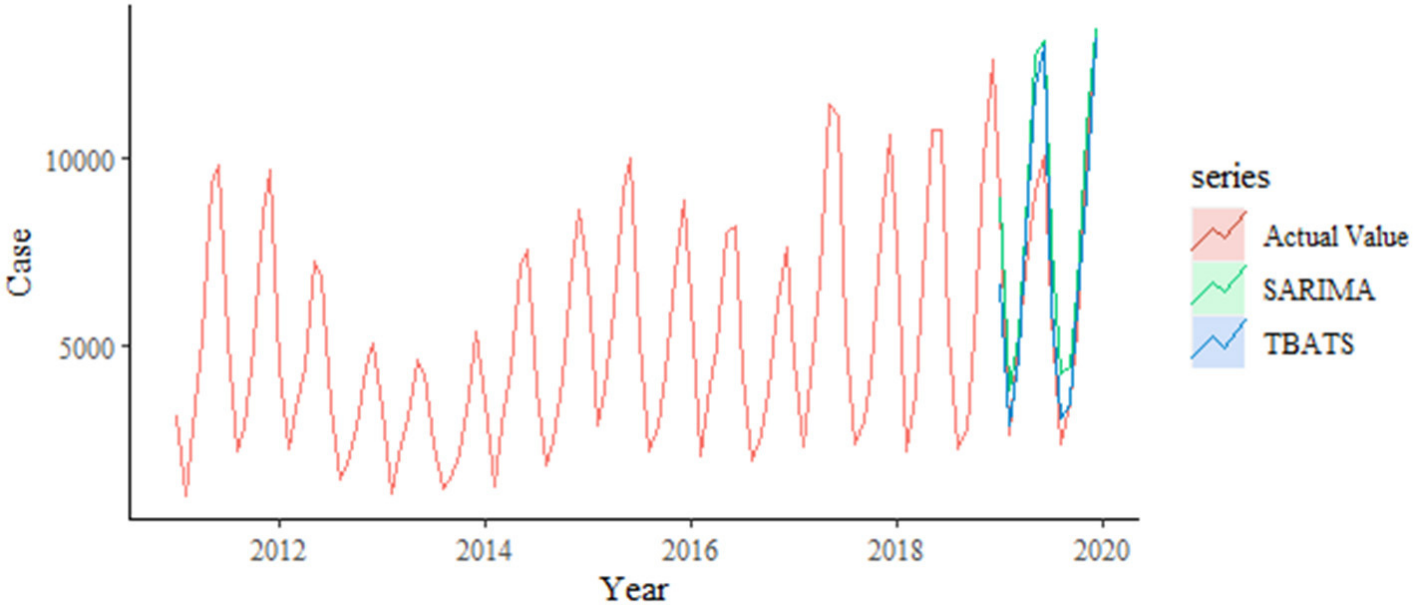
Comparison of SARIMA and TBATS models prediction fit.

The results of the model performance metrics for SARIMA (2, 1, 2) (0, 1, 1)[_12]_ and TBATS (0.003, {0, 0}, 0.822, { <12, 5>}) were investigated. The performances of the two models were compared in terms of both simulation and prediction, and the results showed that the RMSE and MAPE measures of the TBATS (0.003, {0, 0}, 0.822, { <12, 5>}) model were lower than those of the SARIMA (2, 1, 2) (0, 1, 1)_[12]_ model (Table 2). Therefore, the TBATS (0.003, {0, 0}, 0.822, { <12, 5>}) model worked better (Figure 5).

**Table 2:** Comparison of the model fitting effect.

**Model**	**RMSE**	**MAPE**
TBATS	1,423.6	0.14
SARIMA	1,705.65	0.25

Based on the comparison of the above models, we found that the TBATS (0.003, {0, 0}, 0.822,{ <12, 5>}) model had a better level of predictive accuracy, with incidence rates close to their expected levels in years. Therefore, this study used the new TBATS (0.002, {0, 0}, 0.801, { <12, 5>}) model from 2011 to 2019 to predict the incidence of scarlet fever during the 2020 COVID-19 pandemic. The final prediction model showed that the number of scarlet fever cases that were expected to occur in mainland China in 2020 was 89,354, whereas the actual number of cases was 17,206, with a total of 72,148 averted cases, showing an unprecedented decline. The actual number of cases that occurred in January 2020 showed the lowest relative decrease of only 11.47%, which was similar to the expected value (Table 3). The incidence of scarlet fever plummeted from February until May, when it dropped to the lowest level.

**Table 3:** Comparison between actual and expected incidence in 2020, and the relative reduction in average incidence from 2016 to 2019.

**Month**	**2020**	**Expect value**	**Relative reduction**	**2020**	**2016–2019**	**Relative reduction**
January	6,352	7,175	11.47%	6,352	6,797	6.54%
February	580	3,002	80.68%	580	2,290	74.67%
March	444	4,822	90.79%	444	4,386	89.88%
April	442	8,239	94.63%	442	6,377	93.07%
May	562	11,687	95.19%	562	9,801	94.27%
June	677	13,018	94.80%	677	10,022	93.24%
July	789	6,167	87.21%	789	5,201	84.83%
August	763	3,047	74.96%	763	2,235	65.86%
September	877	3,455	74.61%	877	2,962	70.39%
October	1,102	6,117	81.98%	1,102	4,663	76.37%
November	1,925	9,264	79.22%	1,925	8,547	77.48%
December	2,693	13,362	79.85%	2,693	10,970	75.45%
Total	17,206	89,354	80.74%	17,206	74,248	76.83%

**Figure 6:**
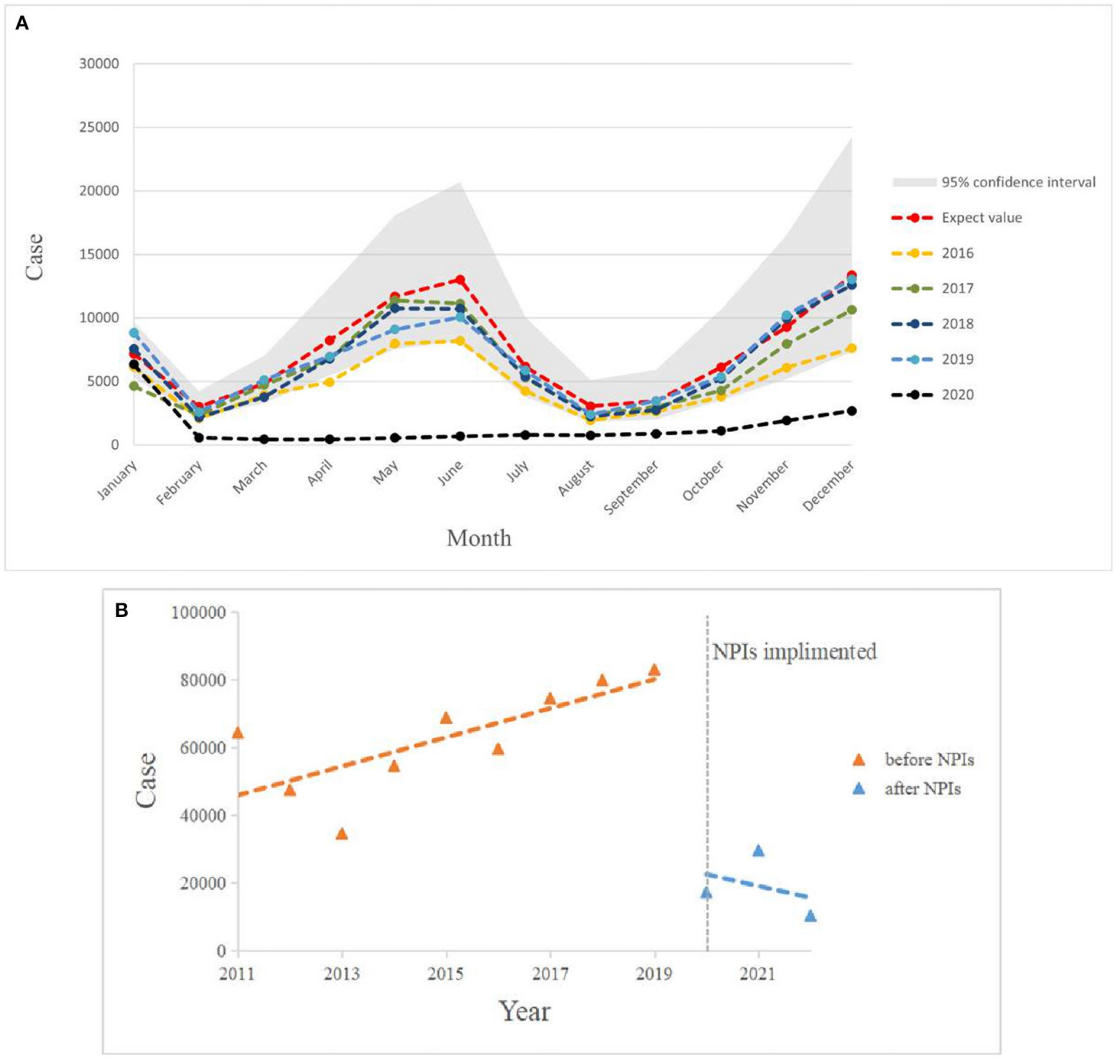
**(A)** Graph of monthly scarlet fever incidence trends in mainland China from 2016 to 2020 compared with expected values in 2020. **(B)** Incidence of scarlet fever before and after NPIs intervention in China, January 2011 to May 2022.

In Figure 6A, the expected incidence rate during the continuing spread of COVID-19 in 2020 differed significantly from the actual incidence rate trend, with the forecast showing a large fluctuating trend while the actual incidence rate showed a low and stable trend. Compared to the average number of cases in 2016–2019, the number of cases during the COVID-19 pandemic decreased by 76.83%, which is a much lower rate than the historical average incidence rate (Table 3). Surprisingly, there was a downward trend in the number of cases, wherein an increase should have occurred in March. Even in May and June, when scarlet fever was the most prevalent, the number of cases did not increase significantly, but instead decreased by ~95% compared to the predicted value and showed a flat trend. We also found that the dual seasonality of scarlet fever during COVID-19 pandemic was no longer prominent, and the proportion of scarlet fever cases, although increasing again in November, remained below the expected level.

Figure 6B shows that the total annual incidence of scarlet fever in China showed a steady increase from 2011 to 2019 (β1 = 4396, *P* < 0.05) and an immediate decrease in 2020 (β2 = −61526, *P* < 0.05). The post-intervention change was not statistically significant (Table 4).

**Table 4:** Interrupted time series analysis of the annual number of scarlet fever cases in mainland China from January 2011 to May 2022.

**Series**	**Estimate**	**S.E**.	***t***	***P*-value**
Intercept	40,643	7,588	5.357	<0.001
Preintervention trend	4,396	1,355	3.245	0.012
Intervening variable	−61,526	12,946	−4.753	0.001
Postintervention	−7,981	8,081	0.988	0.352

Table 5 shows a significant negative correlation between the number of scarlet fever episodes and each NPIs in mainland China (*P* < 0.05). By ridge regression analysis, the *k*-value in this study was 0.103, and ANOVA result was significant (*P* < 0.05). As seen in Table 6, among the eight NPIs, only cancellation of public events (c3) was statistically significant (*P* = 0.045).

**Table 5:** Correlation of the number of scarlet fever cases with each NPI from January 2011 to May 2022.

		**Cases**	**c1**	**c2**	**c3**	**c4**	**c5**	**c6**	**c7**	**c8**
Cases	*r*	1	−0.449**	−0.454**	−0.460**	−0.460**	−0.384**	−0.437**	−0.453**	−0.444**
c1	*r*	−0.449**	1	0.964**	0.978**	0.977**	0.838**	0.948**	0.974**	0.910**
c2	*r*	−0.454**	0.964**	1	0.982**	0.981**	0.836**	0.979**	0.986**	0.914**
c3	*r*	−0.460**	0.978**	0.982**	1	0.998**	0.838**	0.958**	0.989**	0.956**
c4	*r*	−0.460**	0.977**	0.981**	0.998**	1	0.836**	0.957**	0.986**	0.956**
c5	*r*	−0.384**	0.838**	0.836**	0.838**	0.836**	1	0.866**	0.859**	0.796**
c6	*r*	−0.437**	0.948**	0.979**	0.958**	0.957**	0.866**	1	0.970**	0.900**
c7	*r*	−0.453**	0.974**	0.986**	0.989**	0.986**	0.859**	0.970**	1	0.929**
c8	*r*	−0.444^**^	0.910^**^	0.914^**^	0.956^**^	0.956^**^	0.796^**^	0.900^**^	0.929^**^	1

Pearson correlation coefficient is “*r*”.

** *P* < 0.01.

Eight specific NPIs in OxCGRT's containment and closure policy: school closures (c1), workplace closures (c2), cancellation of public events (c3), restrictions on public gatherings (c4), closures of public transport (c5), stay-at-home requirements (c6), restrictions on internal movements (c7), and international travel controls (c8).

**Table 6:** Ridge regression analysis between the number of scarlet fever cases and each NPI in mainland China from January 2011 to May 2022.

	**Coeff**	**S.E**.	***t***	***p***	**Std. coef**	**VIF**
c1	−497.895	731.038	−0.681	0.497	−0.060	1.309
c2	−670.791	582.133	−1.152	0.251	−0.078	0.777
c3	−638.506	315.099	−2.026	0.045	−0.085	0.297
c4	−609.591	368.837	−1.653	0.101	−0.081	0.404
c5	−67.180	1,025.133	−0.066	0.948	−0.006	1.628
c6	−83.115	758.895	−0.110	0.913	−0.010	1.316
c7	−410.545	498.746	−0.823	0.412	−0.052	0.676
c8	−742.939	843.410	−0.881	0.380	−0.087	1.641
Constant	5,241.198	916.078	5.721	0.000	0.000	0.000

Eight specific NPIs in OxCGRT's containment and closure policy: school closures (c1), workplace closures (c2), cancellation of public events (c3), restrictions on public gatherings (c4), closures of public transport (c5), stay-at-home requirements (c6), restrictions on internal movements (c7), and international travel controls (c8).

## 4. Discussion

In brief, this study revealed the demographic and spatiotemporal distribution of the incidence of scarlet fever and patient characteristics in China from 2011 to 2017, with a fluctuating upward trend toward recent years. The comparison of the predicted and actual values of scarlet fever incidence in mainland China in 2020 also revealed an unprecedented downward trend, improving ~80% of infections among the susceptible population. We confirmed that the comprehensive NPIs implemented during the COVID-19 pandemic led to a reduction in the number of scarlet fever cases; in particular, the cancellation of public events had the most significant effect.

Based on the incidence of scarlet fever in mainland China from 2011 to 2017, the following conclusions can be drawn: scarlet fever is predominantly a childhood disease in China, and boys are more likely to contract scarlet fever than girls, which is consistent with the results of other studies (38). Children are prone to aggregate scarlet fever infection due to factors such as their weak resistance and high risk of exposure to the virus during school days. Furthermore, boys are associated with being more active than girls during the school year, thus increasing the risk of illness to some extent. That the incidence of scarlet fever reached a new peak in 2017 may be closely related to the effective implementation of the two-child policy (16).

In addition, scarlet fever has two seasonal peaks in mainland China each year, when children are in school, with cases decreasing during winter, and during summer holidays. The results of this study show that the incidence of scarlet fever varies significantly in different regions, which is consistent with other studies (5, 15). High incidence concentrations are mainly in the north, such as Shandong, Liaoning, Heilongjiang, Beijing, and Hebei, and previous studies have shown that this may be positively correlated with mean temperature and mean relative humidity (39). In addition, a study in Beijing showed that the incidence of scarlet fever was positively correlated with the number of hours of sunshine. The low incidence clusters are mainly in the south, such as Jiangxi, Guangxi, Guizhou, Hunan, and Hubei, and the incidence is especially low in winter, which may be related to the low sunshine during the rainy winter months. The causes of infectious diseases are complex and are not caused by a single meteorological factor, but are also closely related to social factors and pathogenetic characteristics (40). Therefore, school-based preventive and control measures are particularly important for preventing scarlet fever, such as paying attention to personal hygiene in schools, increasing the frequency of disinfection in schools, and strengthening exercises to enhance students' physical fitness. In addition, health departments should pay more attention to the surveillance, prevention, and control of infectious diseases; formulate scientific public health policies; and implement effective interventions to control infectious diseases and protect the children.

For the choice of the prediction model, the TBATS model was found to have higher predictive performance and was more suitable for predicting the incidence of scarlet fever in China. Interestingly, we conclude that the expected incidence of scarlet fever in mainland China in 2020 showed an opposite trend to the actual incidence. Nearly 90,000 cases of scarlet fever were predicted to occur in mainland China in 2020, and the implementation of NPIs in the context of COVID-19 may have prevented more scarlet fever infections. Further exploration using ITSA showed a tendency toward a decrease trend in the total annual incidence of scarlet fever in China from January 2020 to May 2022, indicating that comprehensive NPIs achieved better results. This is largely due to the government's prevention and control policies as well as voluntary behavioral changes by individuals with reduced exposure risk, hospital visits, and exposure to counseling, which have greatly reduced the likelihood of disease transmission. For example, on January 20, 2020, the National Health and Wellness Commission included COVID-19 in the management of statutory category B infectious diseases, and in February, 2020, the State Council issued a notice on the prevention and control of COVID-19 in children and pregnant women. Therefore, this may be an important reason why scarlet fever incidence was not increased, but showed a downward trend in March, 2020. Thereafter, it did not show an increase in May and June, the strongest months of the season, in accordance with previous trends. This may be closely related to the importance of school closures in mitigating the spread of seasonal infections, similar to the findings of other related studies (41). Besides focusing on susceptible populations, it is also important to note that scarlet fever is an infectious disease that is prone to aggregate transmissions, and that, of the eight specific NPIs, the cancellation of public events had the most prominent impact in this study. In addition, a global study confirmed the cancellation of public events as an effective intervention to reduce COVID-19 infection rates (42). Therefore, in the absence of a vaccine or effective drugs for scarlet fever, reducing the risk of transmission and preventing infection may be the best way to reduce the number of scarlet fever cases. In the future, combination of vaccines, drug therapy, and NPIs should be considered as a most effective preventive measure.

Despite these findings, some limitations of our study should be mentioned. First, the data are not reported as individual case data, and daily data may be subject to error. Second, the decline in the age structure and regional distribution of scarlet fever due to COVID-19 was not explored because demographic and geographic distribution data were unavailable in 2020. Third, this study utilized two prediction models for scarlet fever, and their applicability to other diseases remains unexplored. In future studies, if daily scarlet fever data are available, it is necessary to conduct an in-depth analysis of the effectiveness of each NPI and further refine the model for the study of other diseases.

## 5. Conclusion

Scarlet fever poses a continuous threat to children in China, especially in the northern region, and it exhibits bimodal seasonal patterns. The TBATS model predicted a higher level of scarlet fever in China than the SARIMA model, showing that more than 80% of infections in susceptible populations was managed under the COVID-19 pandemic prevention policy. Strict NPIs have a positive impact on the prevention of scarlet fever, with the cancellation of public events having the most significant impact. This suggests that government policymakers need to maintain the use of different types of NPIs to prevent scarlet fever in the future, with a focus on vaccine development and drug treatment. In addition, data limitations suggest the need to still explore the impact of scarlet fever in different regions in the future.

## Data availability statement

Publicly available datasets were analyzed in this study. This data can be found here: https://www.phsciencedata.cn/Share/index.jsp, http://www.nhc.gov.cn/wjw/index.shtml, https://ourworldindata.org/covid-stringency-index.

## Author contributions

YM and SG devised the concept, formal analysis, and writing of the original draft. QW and HL were responsible for the funding acquisition and project administration. ZK, LS, BZ, YC, and LG were involved in the collection of resources to help write the manuscript. YL and LL designed the analyses framework. MJ was responsible for data analyses. YH, NN, and HS assisted with the writing of review and editing. All authors contributed to the article and approved the submitted version.

## Footnotes

^1^*The China Public Health Science Data Center*. Available from: https://www.phsciencedata.cn/Share/index.jsp.

^2^*The National Heath Commission of the People's Republic of China*. Available from: http://www.nhc.gov.cn/wjw/index.shtml.

^3^*Our Word in Data*. Available from: https://ourworldindata.org/covid-stringency-index.

## References

[1] Wong SSY Yuen KY. The comeback of scarlet fever. EBioMedicine. (2018) 28:7–8. 10.1016/j.ebiom.2018.01.030 29396303 PMC5835575

[2] Miller KM Carapetis JR Van Beneden CA Cadarette D Daw JN Moore HC. The global burden of sore throat and group a *Streptococcus pharyngitis*: a systematic review and meta-analysis. EClinicalMedicine. (2022) 48:101458. 10.1016/j.eclinm.2022.101458 35706486 PMC9124702

[3] Lozano R Naghavi M Foreman K Lim S Shibuya K Aboyans V. Global and regional mortality from 235 causes of death for 20 age groups in 1990 and 2010: a systematic analysis for the global burden of disease study 2010. Lancet. (2012) 380:2095–128. 10.1016/S0140-6736(12)61728-0 23245604 PMC10790329

[4] Carapetis JR Steer AC Mulholland EK Weber M. The global burden of group a streptococcal diseases. Lancet Infect Dis. (2005) 5:685–94. 10.1016/S1473-3099(05)70267-X 16253886

[5] You Y Davies MR Protani M McIntyre L Walker MJ Zhang J. Scarlet fever epidemic in China caused by *Streptococcus pyogenes* serotype M12: epidemiologic and molecular analysis. EBioMedicine. (2018) 28:128–35. 10.1016/j.ebiom.2018.01.010 29342444 PMC5835554

[6] Zhang Q Liu W Ma W Shi Y Wu Y Li Y. Spatiotemporal epidemiology of scarlet fever in Jiangsu Province, China, 2005-2015. BMC Infect Dis. (2017) 17:596. 10.1186/s12879-017-2681-5 28854889 PMC5576110

[7] Rocco R Benedetti L Escudero G Jordán F. Hydrops of the gallbladder and hepatitis associated with scarlet fever. Acta Gastroenterol Latinoam. (2010) 40:61–4. 20446398

[8] Slebioda Z Mania-Końsko A Dorocka-Bobkowska B. Scarlet fever - a diagnostic challenge for dentists and physicians: a report of 2 cases with diverse symptoms. Dental Med Probl. (2020) 57:455–9. 10.17219/dmp/125574 33448168

[9] Duncan SR Scott S Duncan CJ. Modelling the dynamics of scarlet fever epidemics in the 19th century. Eur J Epidemiol. (2000) 16:619–26. 10.1023/A:1007645110006 11078118

[10] Duncan CJ Duncan SR Scott S. The dynamics of scarlet fever epidemics in England and Wales in the 19th century. Epidemiol Infect. (1996) 117:493–9. 10.1017/S0950268800059161 8972674 PMC2271647

[11] Katz AR Morens DM. Severe streptococcal infections in historical perspective. Clin Infect Dis. (1992) 14:298–307. 10.1093/clinids/14.1.298 1571445

[12] Park DW Kim SH Park JW Kim MJ Cho SJ Park HJ. Incidence and characteristics of scarlet fever, South Korea, 2008-2015. Emerg Infect Dis. (2017) 23:658–61. 10.3201/eid2304.160773 28322696 PMC5367408

[13] Lamagni T Guy R Chand M Henderson KL Chalker V Lewis J. Resurgence of scarlet fever in England, 2014-16: a population-based surveillance study. Lancet Infect Dis. (2018) 18:180–7. 10.1016/S1473-3099(17)30693-X 29191628

[14] You Y Qin Y Walker MJ Feng L Zhang J. Increased incidence of scarlet fever—China, 1999-2018. China CDC Wkly. (2019) 1:63. 10.46234/ccdcw2019.019 34594606 PMC8393181

[15] Liu Y Chan TC Yap LW Luo Y Xu W Qin S. Resurgence of scarlet fever in China: a 13-year population-based surveillance study. Lancet Infect Dis. (2018) 18:903–12. 10.1016/S1473-3099(18)30231-7 29858148 PMC7185785

[16] Zeng Y Hesketh T. The effects of China's universal two-child policy. Lancet. (2016) 388:1930–8. 10.1016/S0140-6736(16)31405-2 27751400 PMC5944611

[17] Jiang F Wei T Hu X Han Y Jia J Pan B. The association between ambient air pollution and scarlet fever in Qingdao, China, 2014-2018: a quantitative analysis. BMC Infect Dis. (2021) 21:987. 10.1186/s12879-021-06674-8 34548016 PMC8456591

[18] Kim DH Nguyen TM Kim JH. Infectious respiratory diseases decreased during the Covid-19 pandemic in South Korea. Int J Environ Res Public Health. (2021) 18:2. 10.3390/ijerph18116008 34205018 PMC8199908

[19] Sakamoto H Ishikane M Ueda P. Seasonal influenza activity during the SARS-CoV-2 outbreak in Japan. JAMA. (2020) 323:1969–71. 10.1001/jama.2020.6173 32275293 PMC7149351

[20] Redlberger-Fritz M Kundi M Aberle SW Puchhammer-Stöckl E. Significant impact of nationwide SARS-CoV-2 lockdown measures on the circulation of other respiratory virus infections in Austria. J Clin Virol. (2021) 137:104795. 10.1016/j.jcv.2021.104795 33761423 PMC7962988

[21] Geng MJ Zhang HY Yu LJ Lv CL Wang T Che TL. Changes in notifiable infectious disease incidence in China during the Covid-19 pandemic. Nat Commun. (2021) 12:6923. 10.1038/s41467-021-27292-7 34836947 PMC8626444

[22] Li Y Campbell H Kulkarni D Harpur A Nundy M Wang X. The Temporal association of introducing and lifting non-pharmaceutical interventions with the time-varying reproduction number (R) of SARS-CoV-2: a modelling study across 131 countries. Lancet Infect Dis. (2021) 21:193–202. 10.1016/S1473-3099(20)30785-4 33729915 PMC7581351

[23] Pan A Liu L Wang C Guo H Hao X Wang Q. Association of public health interventions with the epidemiology of the Covid-19 outbreak in Wuhan, China. JAMA. (2020) 323:1915–23. 10.1001/jama.2020.6130 32275295 PMC7149375

[24] Hagens A Cordova-Pozo K Postma M Wilschut J Zino L van der Schans J. Reconstructing the effectiveness of policy measures to avoid next-wave Covid-19 infections and deaths using a dynamic simulation model: implications for health technology assessment. Front Med Technol. (2021) 3:666581. 10.3389/fmedt.2021.666581 35156083 PMC8825500

[25] Song S Li Q Shen L Sun M Yang Z Wang N. From outbreak to near disappearance: how did non-pharmaceutical interventions against Covid-19 affect the transmission of influenza virus? Front Public Health. (2022) 10:863522. 10.3389/fpubh.2022.863522 35425738 PMC9001955

[26] Lai S Ruktanonchai NW Zhou L Prosper O Luo W Floyd JR. Effect of non-pharmaceutical interventions to contain Covid-19 in China. Nature. (2020) 585:410–3. 10.1038/s41586-020-2293-x 32365354 PMC7116778

[27] Song X Xiao J Deng J Kang Q Zhang Y Xu J. Time series analysis of influenza incidence in Chinese provinces from 2004 to 2011. Medicine. (2016) 95:e3929. 10.1097/MD.0000000000003929 27367989 PMC4937903

[28] Liang F Guan P Wu W Huang D. Forecasting influenza epidemics by integrating internet search queries and traditional surveillance data with the support vector machine regression model in Liaoning, from 2011 to 2015. PeerJ. (2018) 6:e5134. 10.7717/peerj.5134 29967755 PMC6022725

[29] Wang Y Xu C Wang Z Yuan J. Seasonality and trend prediction of scarlet fever incidence in Mainland China from 2004 to 2018 using a hybrid Sarima-Narx Model. PeerJ. (2019) 7:e6165. 10.7717/peerj.6165 30671295 PMC6339779

[30] Cherrie MPC Nichols G Iacono GL Sarran C Hajat S Fleming LE. Pathogen seasonality and links with weather in England and Wales: a big data time series analysis. BMC Public Health. (2018) 18:1067. 10.1186/s12889-018-5931-6 30153803 PMC6114700

[31] De Livera AM Hyndman RJ Snyder RD. Forecasting time series with complex seasonal patterns using exponential smoothing. J Am Stat Assoc. (2011) 106:1513–27. 10.1198/jasa.2011.tm0977130475836

[32] Taylor JW. Short-term electricity demand forecasting using double seasonal exponential smoothing. J Operat Res Soc. (2017) 54:799–805. 10.1057/palgrave.jors.2601589

[33] Xiao Y Li Y Li Y Yu C Bai Y Wang L. Estimating the long-term epidemiological trends and seasonality of hemorrhagic fever with renal syndrome in China. Infect Drug Resist. (2021) 14:3849–62. 10.2147/IDR.S325787 34584428 PMC8464322

[34] Sakizadeh M. Spatiotemporal variations and characterization of the chronic cancer risk associated with benzene exposure. Ecotoxicol Environ Saf. (2019) 182:109387. 10.1016/j.ecoenv.2019.109387 31302332

[35] Li Z Wang Z Song H Liu Q He B Shi P. Application of a hybrid model in predicting the incidence of tuberculosis in a Chinese population. Infect Drug Resist. (2019) 12:1011–20. 10.2147/IDR.S190418 31118707 PMC6501557

[36] Wang Y Xu C Zhang S Yang L Wang Z Zhu Y. Development and evaluation of a deep learning approach for modeling seasonality and trends in hand-foot-mouth disease incidence in Mainland China. Sci Rep. (2019) 9:8046. 10.1038/s41598-019-44469-9 31142826 PMC6541597

[37] Wang Y Xu C Ren J Wu W Zhao X Chao L. Secular seasonality and trend forecasting of tuberculosis incidence rate in Chinausing the advanced error-trend-seasonal framework. Infect Drug Resist. (2020) 13:733–47. 10.2147/IDR.S238225 32184635 PMC7062399

[38] Rao HX Li DM Zhao XY Yu J. Spatiotemporal clustering and meteorological factors affected scarlet fever incidence in Mainland China from 2004 to 2017. Sci Total Environ. (2021) 777:146145. 10.1016/j.scitotenv.2021.146145 33684741

[39] Huo A Zhao D Fang L Cao W. Relationship between main aspiratory infectious diseases and meteorological factors in North China. Chin Med Herald. (2011) 8:153–6.

[40] Zhong WQ ZY Su Y Bai J Dong R Zheng XH. Analysis of the association between scarlet fever and meteorological factors in Heilongjiang Province from 2010 to 2020. Chin J Bioinformat. (2021) 19:4. 10.12113/202107001

[41] Cauchemez S Valleron AJ Boëlle PY Flahault A Ferguson NM. Estimating the impact of school closure on influenza transmission from sentinel data. Nature. (2008) 452:750–4. 10.1038/nature06732 18401408

[42] Sun J Zheng Y Liang W Yang Z Zeng Z Li T. Quantifying the effect of public activity intervention policies on Covid-19 pandemic containment using epidemiologic data from 145 countries. Value Health. (2022) 25:699–708. 10.1016/j.jval.2021.10.007 35500944 PMC8651490

