# Supplementary material for: Epidemiological trend in scarlet fever incidence in China during the COVID-19 pandemic: A time series analysis
Source: Front Public Health. 2022 Dec 15;10:923318. doi: 10.3389/fpubh.2022.923318 (PMC9799716; doi:10.3389/fpubh.2022.923318)
Supplement: Supplementary file 1 [file Table_1.DOCX]

**Supplementary material**

**Supplementary Table 1** Sensitivity analysis of SARIMA related parameters.

| Parameters | Coefficients | S.E. | AIC | Ljung-Box | |
| --- | --- | --- | --- | --- | --- |
|  |  |  |  | Statistics | p |
| SARIMA(0,1,0)(0,1,1)_[12]_ p=0,q=0 | -0.458 | 0.160 | 1378.800 | 5.764 | 0.016 |
| SARIMA(0,1,1)(0,1,1)_[12]_ p=0,q=1 | -0.487 | 0.147 | 1369.580 | 0.497 | 0.481 |
| SARIMA(0,1,2)(0,1,1)_[12]_ p=0,q=2 | -0.472 | 0.142 | 1364.310 | 0.756 | 0.385 |
| SARIMA(0,1,3)(0,1,1)_[12]_ p=0,q=3 | -0.494 | 0.140 | 1363.210 | 0.028 | 0.867 |
| SARIMA(1,1,0)(0,1,1)_[12]_ p=1,q=0 | -0.458 | 0.145 | 1361.590 | 0.055 | 0.814 |
| SARIMA(1,1,1)(0,1,1)_[12]_ p=1,q=1 | -0.467 | 0.146 | 1368.720 | 0.014 | 0.907 |
| SARIMA(1,1,2)(0,1,1)_[12]_ p=1,q=2 | -0.491 | 0.140 | 1362.720 | 0.010 | 0.920 |
| SARIMA(1,1,3)(0,1,1)_[12]_ p=1,q=3 | -0.495 | 0.140 | 1364.510 | 4.321 | 0.995 |
| SARIMA(2,1,0)(0,1,1)_[12]_ p=2,q=0 | -0.509 | 0.140 | 1363.900 | 0.642 | 0.423 |
| SARIMA(2,1,1)(0,1,1)_[12]_ p=2,q=1 | -0.530 | 0.136 | 1358.930 | 0.214 | 0.644 |
| SARIMA(2,1,2)(0,1,1)_[12]_ p=2,q=2 | -0.551 | 0.135 | 1356.480 | 0.002 | 0.966 |
| SARIMA(2,1,3)(0,1,1)_[12]_ p=2,q=3 | -0.522 | 0.140 | 1357.500 | 0.136 | 0.713 |
| SARIMA(3,1,0)(0,1,1)_[12]_ p=3,q=0 | -0.536 | 0.137 | 1361.980 | 0.693 | 0.405 |
| SARIMA(3,1,1)(0,1,1)_[12]_ p=3,q=1 | -0.537 | 0.135 | 1360.210 | 0.114 | 0.736 |
| SARIMA(3,1,2)(0,1,1)_[12]_ p=3,q=2 | -0.508 | 0.137 | 1361.310 | 0.380 | 0.538 |
| SARIMA(3,1,3)(0,1,1)_[12]_ p=3,q=3 | -0.519 | 0.135 | 1361.230 | 0.143 | 0.706 |
